# Modulation and Characterization of Wax-Based Olive Oil Organogels in View of Their Application in the Food Industry

**DOI:** 10.3390/gels7010012

**Published:** 2021-01-28

**Authors:** Pedro M. Silva, Artur J. Martins, Luiz H. Fasolin, António A. Vicente

**Affiliations:** 1 Centre of Biological Engineering, Campus de Gualtar, University of Minho, 4710-057 Braga, Portugal; pedro.silva@ceb.uminho.pt; 2 International Iberian Nanotechnology Laboratory, Food Processing and Nutrition Group, Av. Mestre José Veiga s/n, 4715-330 Braga, Portugal; artur.martins@inl.int; 3 Department of Food Engineering, School of Food Engineering, University of Campinas—UNICAMP, 13083-862 Campinas, SP, Brazil; lfasolin@unicamp.br

**Keywords:** organogel, gelation, texture, rheology, olive oil, natural waxes

## Abstract

Olive oil has recognized health benefits but lacks structural resilience to act in a similar fashion as do the typically used triglycerides (TAGs) when applied in food manufacturing. Therefore, olive oil structuring is critical to widening its use as a healthier alternative in spreadable products. Foreseeing the development of an application for the food industry, three types of natural waxes were used as organogelators, generating olive oil organogels with distinct properties. Retail-simulated storage conditions were used to mimic real-life industrial and commercial use. Organogel systems were evaluated according to their oxidation stability and textural and rheological properties. Textural and rheological parameters increased in response to increasing gelator concentration, while oxidation values (below 1.5 meq O_2_·kg^−1^) remained within legal limits. Organogels displayed similar textural properties to those of commercially available spreadable products, while displaying a low critical gelation concentration. In short, it was shown that tailoring the physicochemical properties of organogels towards specific applications is possible. The produced organogels showed similar properties to the ones of commercially available spreadable products, revealing favourable oxidative profiles. Therefore, an industrial application can be easily foreseen, building on the natural characteristics of olive oil as a healthier alternative to current spreadable products.

## 1. Introduction

Polyunsaturated natural vegetable or fruit oils are known to have several benefits when compared to saturated and/or trans fats, which are associated to negative effects such as oxidative stress, cancer onset, cardiovascular diseases, increased body weight and insulin resistance [1]. Despite that, such saturated and/or trans fats are generally applied in food products as texture modifiers due to their convenient physical properties, while healthier edible oils are generally found in a liquid state, limiting their applicability. Therefore, modulating the physicochemical properties, by conferring structure to edible oils without changing lipid profile and chemical composition, could overcome this drawback and allow for a greater range of applications [2,3]. Efforts are in place to promote the replacement of saturated fats in foods with healthier unsaturated fats, and because of that, organogel technology has drawn interest as a promising alternative.

Organogelation is considered an advantageous technique to structure liquid oils at room temperature without increasing trans fatty acid content [2,3,4,5,6]. Organogels or oleogels are defined as semi-solid dynamic materials that usually result from the immobilization of an organic liquid in a three-dimensional network formed by a gelling agent that self-assemblies into fibre or plate-like structures either by physical or chemical interactions that prevent solvent flow [7]. In order to provide a three-dimensional structure to these edible natural oils, different groups of gelators can be used, namely, low-molecular-weight organogelators (LMOGs) and polymers [8,9]. Several LMOGs have been explored as gelling agents for vegetable oils [10,11,12]. Polymeric organogels are mainly formed through the cross-linked networks established by covalent interactions, while LMOGs are based mainly on non-covalent interactions (e.g., van der Waals interactions, H-bonding, π-π stacking) [13,14,15].

Natural waxes are some of the most valuable materials for this function due to their excellent oil binding properties, economical value and gelling ability at low concentrations. Additionally, a great number of waxes are approved for use in food products, which is of course a binding condition in the present case in order to allow their application in the food industry [2,16].

Physical properties of organogels are dependent on the molecular structure of both oil and gelling agent used, and on the interaction between them. The use of different oil phases and waxes with different structures may allow for the modulation of such characteristics and development of specifically designed products for food and pharmaceutical industries, namely, products that can act as alternatives to saturated and/or trans fats.

Research regarding organogels is typically conducted with controlled temperature and light exposure, shielding the produced organogels (namely during storage) from both these variables. However, in an industrial setting (both in production and in commercialization), the same type of strict control might not exist, and as such, it is essential to assess how these structured oils might behave in industrial and commercial sets of conditions, with less control over temperature and light exposure during storage [5,11].

Waxes are natural products obtained from animal (e.g., beeswax) or plant sources (e.g., candelilla and carnauba waxes). Beeswax is mainly composed of palmitate, palmitoleate, hydroxypalmitate and oleate esters of long-chain alcohols (C30–32), which amount to roughly 70 to 80% of its total weight. Ethyl esters, aliphatic, unsaturated hydrocarbons, pheromones and terpenoids are also present. Beeswax composition varies according to its source and origin but is roughly estimated to have around 70–71% total esters, 1–1.5% free alcohols, 9–11% free acids and 12–15% of hydrocarbons. Carnauba wax is secreted by the leaves of a Brazilian palm tree (*Copernicia prunifera cerifera*) and contains mainly fatty esters (80–85%), free alcohols (10–15%), acids (3–6%) and hydrocarbons (1–3%), while candelilla wax is produced by small shrubs from Mexico, *Euphorbia cerifera* and *E. antisyphilitica* (*Euphorbiaceae*), and is mainly constituted by hydrocarbons (about 50% of C29 to C33, mainly C31), esters (28–29%), alcohols, free fatty acids (7–9%) and resins (12–14% triterpenoid esters), displaying different physicochemical properties, which will in turn lead to different functional properties [17,18,19,20,21].

Extra virgin olive oil is mostly composed of long-chain triglycerides (LCT) that have been reported to have several important biological properties due to high concentrations (about 70%) of monounsaturated fatty acids (C18:1 oleic acid), also having around 14% saturated fatty acids (12% C16:0 palmitic acid, 2% C18:0 stearic acid) and 8% polyunsaturated fatty acids (C18:2 linoleic acid), among other constituents. In addition, the presence of phenolic compounds also carries benefits to human health [22].

Despite several works assessing the gelling properties of natural waxes, this work assessed the impact of different natural waxes as well as the impact of changes in concentration on the physicochemical properties of olive oil (a commercial LCT) organogels, which were evaluated by phase-contrast microscopy, non-isothermal oscillatory and flow rheology, mechanical properties and oxidative stability. In addition, and most importantly, for the first time, processing and storage conditions close to those seen in industrial and commercial settings (samples prepared and stored at room temperature and subject to light exposure during) are used, and the developed organogels’ resilience to oxidation and storage is tested upon production and subsequently during storage.

## 2. Results and Discussion

### 2.1. Visual Observation and Morphological Analysis

**Figure 1:**
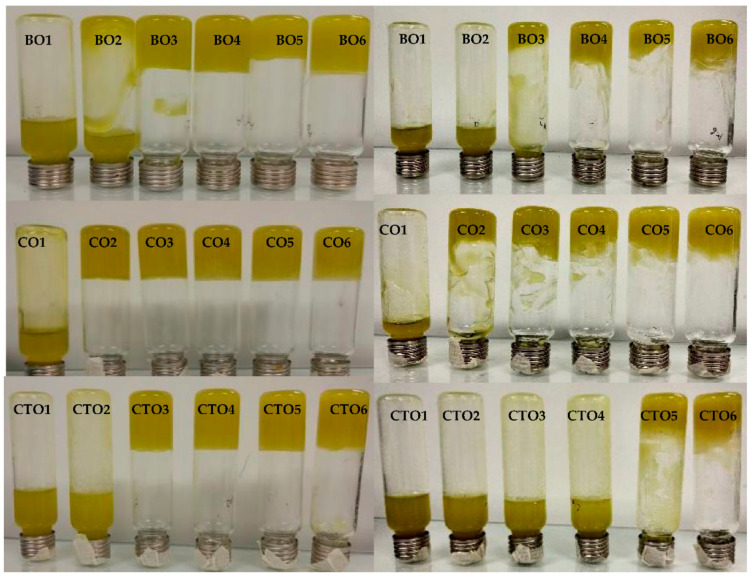
Inversion tests for beeswax (**top**), candelilla (**middle**) and carnauba (**bottom**) organogels. Samples are shown after production (**left**) and after two months of storage (**right**).

Beeswax organogels (BO) were able to self-stand, when inverted, for gelator concentrations higher than 2% (*w*/*w*), while candelilla organogels (CO), did so above 1% (*w*/*w*). On the other hand, carnauba organogels (CTO) showed self-supported gels above 3% (*w*/*w*) concentration (Figure 1). For all gelators, the critical gelation concentration values are in the same range of those found in previous studies, around 3% [5,11,12]. The observed changes in this variable were possibly caused due to the use of extra virgin olive oil exhibiting a long carbon chain, instead of the medium chain triglyceride (MCT) used in the mentioned works. The use of an oil phase with longer chain triglycerides (LCT), in conjugation with lower polarity and higher degree of unsaturation, may have steered to lower gelation concentrations when compared to reports using MCT oils [10,16,23].

After a 2-month period, changes were observed, mainly for carnauba organogels (CTO), which initially showed self-standing ability at 3% (*w*/*w*) and, in the end period, exhibited loss of structure, presenting self-standing ability at 5% (*w*/*w*). BO and CO organogels had a similar behaviour, with self-standing ability at 3 and 2% (*w*/*w*), respectively, diverging to the initial values at 2 and 1% (*w*/*w*), respectively).

**Figure 2:**
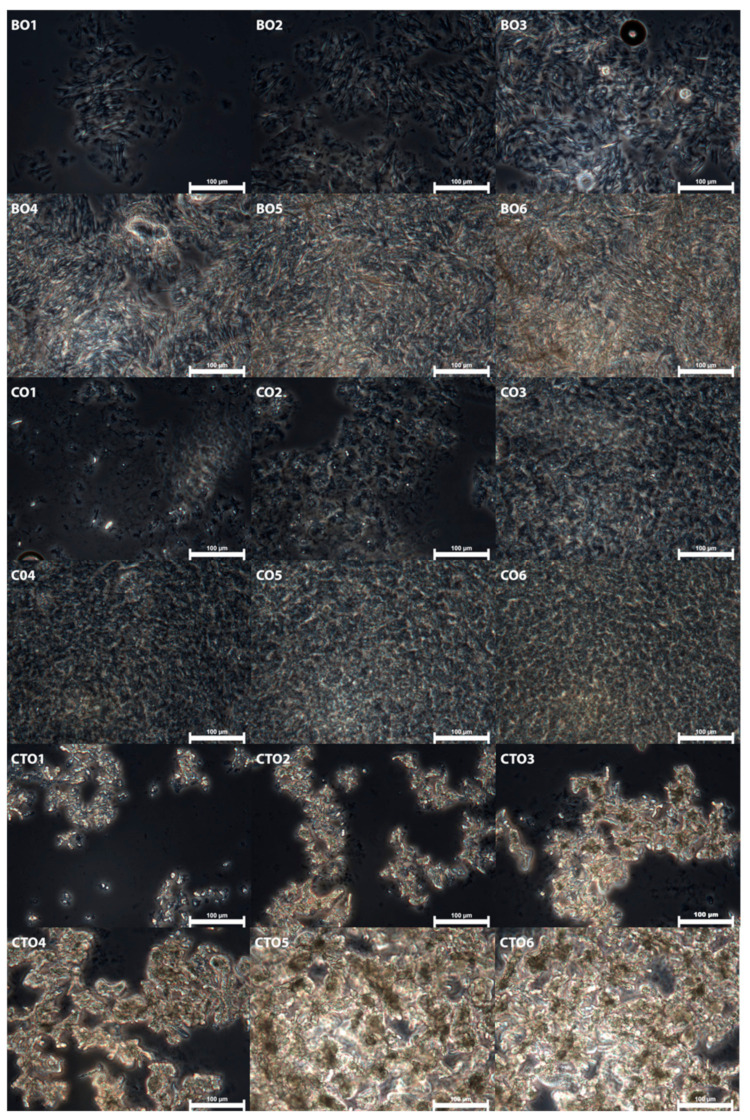
Phase contrast micrographs for all organogels after organogelation.

After achieving gelation, organogels were also evaluated through phase-contrast microscopy to assess the effects of using different waxes and distinct concentrations on the organogel microstructure. These micrographs can be seen in Figure 2. For all organogels, an increase in crystal content with increasing organogelator concentration was observed. A more organized and, thus, stronger 3D structure, which can be observed in the self-standing ability of the organogels, results from higher amounts of crystal agglomerates present when the organogels show self-standing ability. It is known that at lower concentrations, the gelator organogel structure is stabilized through weak interactions such as H-H bonding and polar–polar interactions between components with polar moieties, in addition to weak intermolecular interactions, namely, London dispersion forces, between nonpolar components; while at higher concentrations, above the gelling point of the organogels, the crystal aggregates start to overlap and an interconnect 3D network is formed, leading to the creation of a physical gel [24]. The ability of some of the waxes to create self-standing organogels at lower concentrations might derive from their chemical composition, as well as the morphology of the crystals formed, as waxes with higher hydrocarbon content (HCC) were able to form self-standing organogels at lower concentrations than those with a lower HCC in their composition.

The carbon chain length of the waxes’ chemical components will also influence their gelling ability, with higher chain lengths leading to waxes with a gelling ability at lower concentrations [24]. It is possible to see (Figure 2) that carnauba wax organogels tend to form more crystal clusters, typically with smaller crystals, instead of a more uniform 3D crystal network; as such, higher gelator concentrations are needed to develop a self-standing organogel, and consequently, a higher crystalline mass fraction is needed for gelation to occur [24]. The need for a higher crystalline mass might also be due to carnauba wax’s higher polarity, when compared to other natural waxes. In fact, lower polarities of both waxes and liquids have been linked to better crystallization properties, and other studies have reported on the ability of molecules to arrange in such a fashion to minimize the exposure of their polar groups to the interface of the apolar solvent and its importance in the formation of a stable nucleus [21,25,26,27].

Nevertheless, the chemical composition of waxes also plays a strong role in organogel gelation, with reports of higher wax esters, free fatty acids and HCC leading to stronger, stabler and more consistent (but sometimes more brittle) gels, namely, when a higher wax ester content is present [28,29]. Regarding morphology, beeswax organogels have been described with a “sea urchin”-like morphology, while candelilla organogels tend to form “platelet-like” structures, and carnauba organogels, as previously mentioned, tend to form larger aggregates of small crystals that eventually lead to network formation as a result of overlapping aggregates. The crystal morphology of candelilla and beeswax are more adapted to forming well-defined, self-standing gels, at lower gelator concentrations, as shown in this work [24].

### 2.2. Oxidative Stability

**Figure 3:**
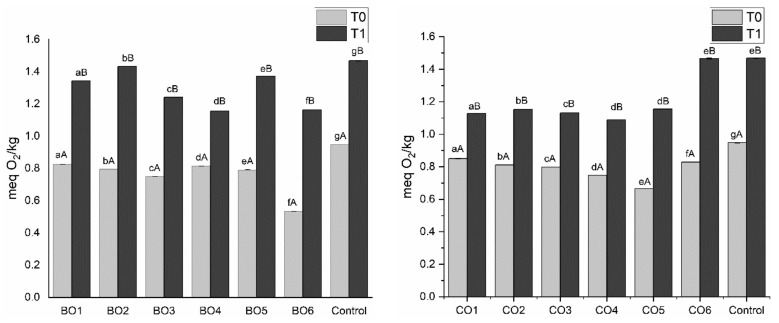

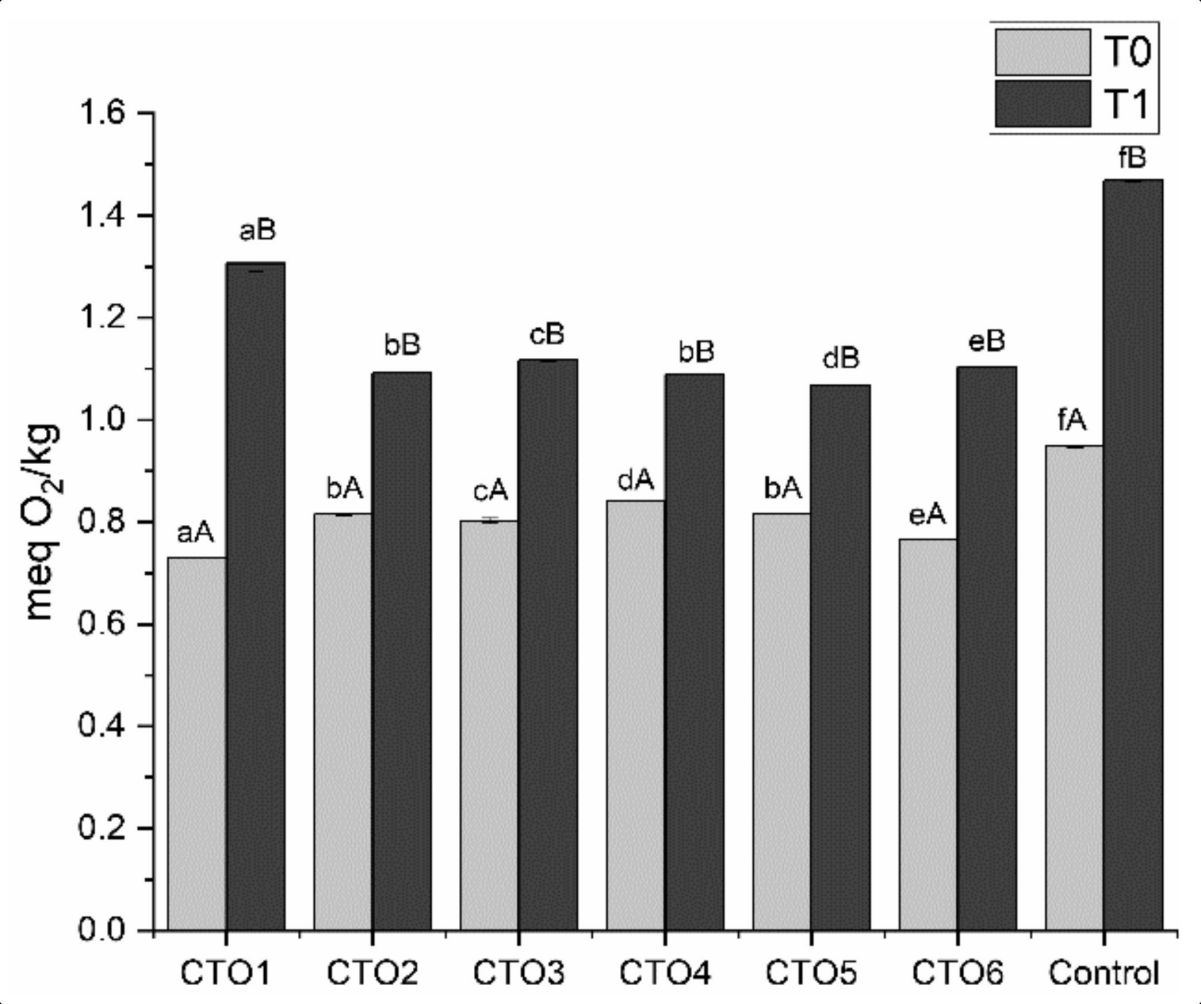
Peroxide analysis results for all organogel and control samples for both times of assessment T0 (0 days) and T1 (2 months). Different small letters indicate statistically significant differences between different concentration within the same organogelator, while different capital letters indicate statistically significant differences between different time assessments of the same organogel (*p* < 0.05).

Given the use of temperature to produce the organogels and the real-life possible use of these products (both at the industry and consumer levels), an analysis of the oxidative stability was necessary. For this reason, samples were stored at room temperature and exposed to light. Peroxide values (PV) were measured upon preparation and after 2 months of storage, to assess the oxidative stability of the samples. These results are shown in Figure 3. According to Tukey’s test, the gelator type, concentration and storage time exerted significant effect (*p* ≤ 0.05) on the PV.

All organogels showed lower PV in comparison to pure olive oil at T0 (after preparation). The organogelation process (high temperatures needed for the solubilization of the natural waxes and to simulate the future use by industry and consumers) apparently did not degrade the antioxidant compounds present in the extra virgin olive oil (0.948 meq O_2_·kg^−1^). Additionally, the increase in soluble solids content (i.e., waxes) helped prevent oxidation, showing lower PV with higher concentrations, except for candelilla organogels, which have a lower PV at 5%. The low PV found could be related to the microcomponents (tocols, polyphenols and sterols) present in the extra virgin olive oil used to prepare the organogels.

During the storage time, there was an increase in PV for all samples used in this work. The pure olive oil after 2 months showed a PV of 1.468 meq O_2_·kg^−1^ that agrees with previous studies [30]. Nevertheless, this result is also well below the defined limit in the European Union for PV of extra virgin olive oil (<20 meq O_2_·kg^−1^) and the limit for peroxide value in edible fats (<10 meq O_2_·kg^−1^), indicating a good oxidative stability of the organogels, considering both the production method and storage conditions of the developed organogels [31,32]. Considering that no external antioxidant was added, we can claim that these products exhibit oxidative stability, due to the olive oil natural properties, during a considerable period of time, allowing their use in commercial applications within the food industry, with no special storage requirements (i.e., shielded from light or refrigerated) needed [3,12].

### 2.3. Mechanical Properties

**Figure 4:**
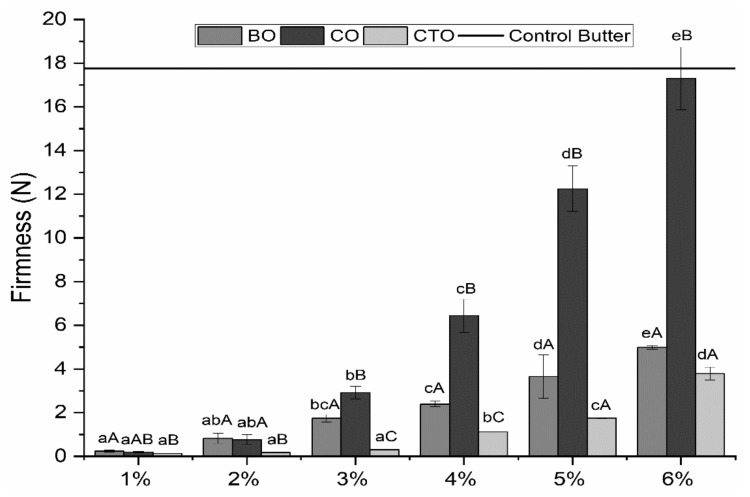

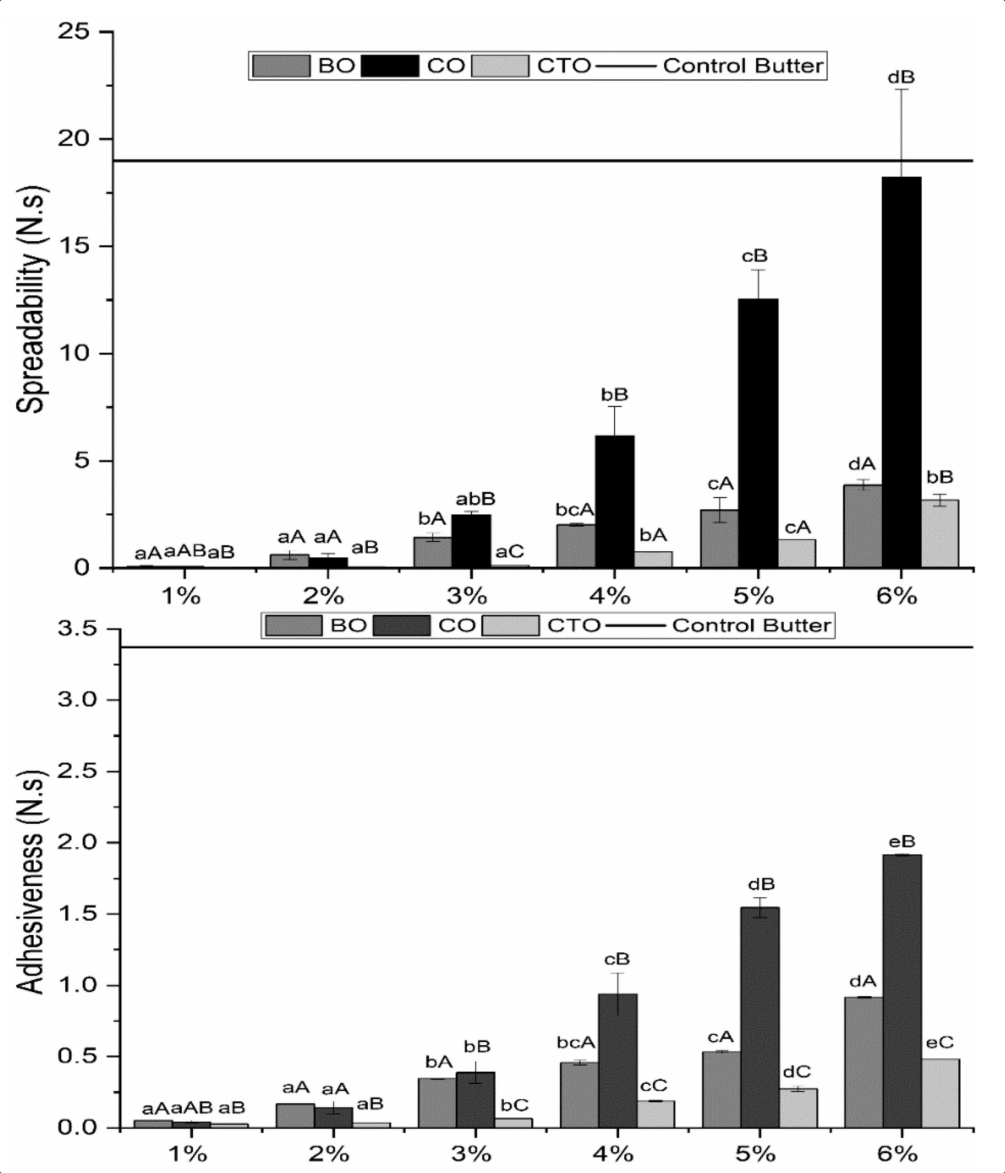
Texture analysis results for firmness (**top**), spreadability (**middle**) and adhesiveness (**bottom**). Different small letters indicate statistically significant differences between different concentrations within the same organogelator, while different capital letters indicate statistically significant differences between different organogels (*p* < 0.05). Extra virgin olive oil obtained values of 0.1035 N, 0.0061 N·s and 0.0199 N·s for firmness, spreadability and adhesiveness, respectively.

Mechanical properties’ results showed that with an increase in organogelator concentration, all the parameters (firmness, spreadability, adhesiveness) increased as well (Figure 4).

At low concentrations, the carnauba wax organogelator was statistically similar (*p* > 0.05) to the control sample of extra virgin olive oil, while at concentrations higher than 3% (*w*/*w*), all organogel samples were statistically different (*p* < 0.05) from the extra virgin olive oil control sample (0.104 N of firmness, 0.006 N·s of spreadability and 0.02 N·s of adhesiveness), indicating a clear difference between structured olive oil (organogels) and normal olive oil. For the beeswax and candelilla waxes, all samples were statistically different (*p* < 0.05) from the control. The values obtained for firmness, spreadability and adhesiveness showed that candelilla organogels had the highest values for all the parameters assessed, followed by the beeswax organogels and carnauba organogels, respectively.

This trend is linked to the hydrocarbon composition of each of the waxes, with candelilla displaying the higher HCC (around 50%), followed by beeswax (12–15%) and carnauba (1–3%), and the importance of HCC on oil gelation has been previously reported [29]. Such a wide range of results, observable in Figure 4, further highlights the possibility of modulation of the desired characteristics of the final product using different organogelators, or by combining different organogelators, which is of particular importance to producing a wide range of possible organogels products for industrial and commercial applications. These results are also in accordance with what was seen in the microscopical analysis (Figure 2), in which a more defined 3D network of crystals was present at higher concentrations of gelator.

A commercial butter was also tested to compare the obtained results to those from a commercially available product and yielded values of 17.76 N for firmness, 18.98 N·s for spreadability and 3.37 N·s for adhesiveness. A control sample of extra virgin olive oil obtained values of 0.1035 N, 0.0061 N·s and 0.0199 N·s for the same parameters, respectively. According to Figure 4, it is possible to observe that CO6 showed similar values to those of butter and, thus, presents itself as a possible healthier alternative, in terms of textural properties, to commercial butter. Some of the more important properties regarding sensory perception of spreadable products are firmness and spreadability, which were found to have a wide range of values, showing tailoring potential for a wide range of spreadable products, including, as seen above, butter [2,3,11,12]. It is important to notice that this result does not exclude the other samples from being used for other food applications, as different products will have different functional needs and properties [30,33,34,35,36]. Alvarez et al. (2011) determined the firmness of mashed potatoes to be around 6 N, Sanders et al. (2014) determined a value of around 9 N for peanut butter, while Hadnadev et al. (2011) found the firmness of edible vegetable fat to be as low as 5 N, and Nikolić et al. (2014) analysed the spreadability of low-fat food spreads made from hull-less pumpkin seed flour and reported values in the same range as those described in this work, thus demonstrating the potential for the applicability of different organogels in the food industry [34,35,36,37].

### 2.4. Rheological Analyses

**Figure 5:**
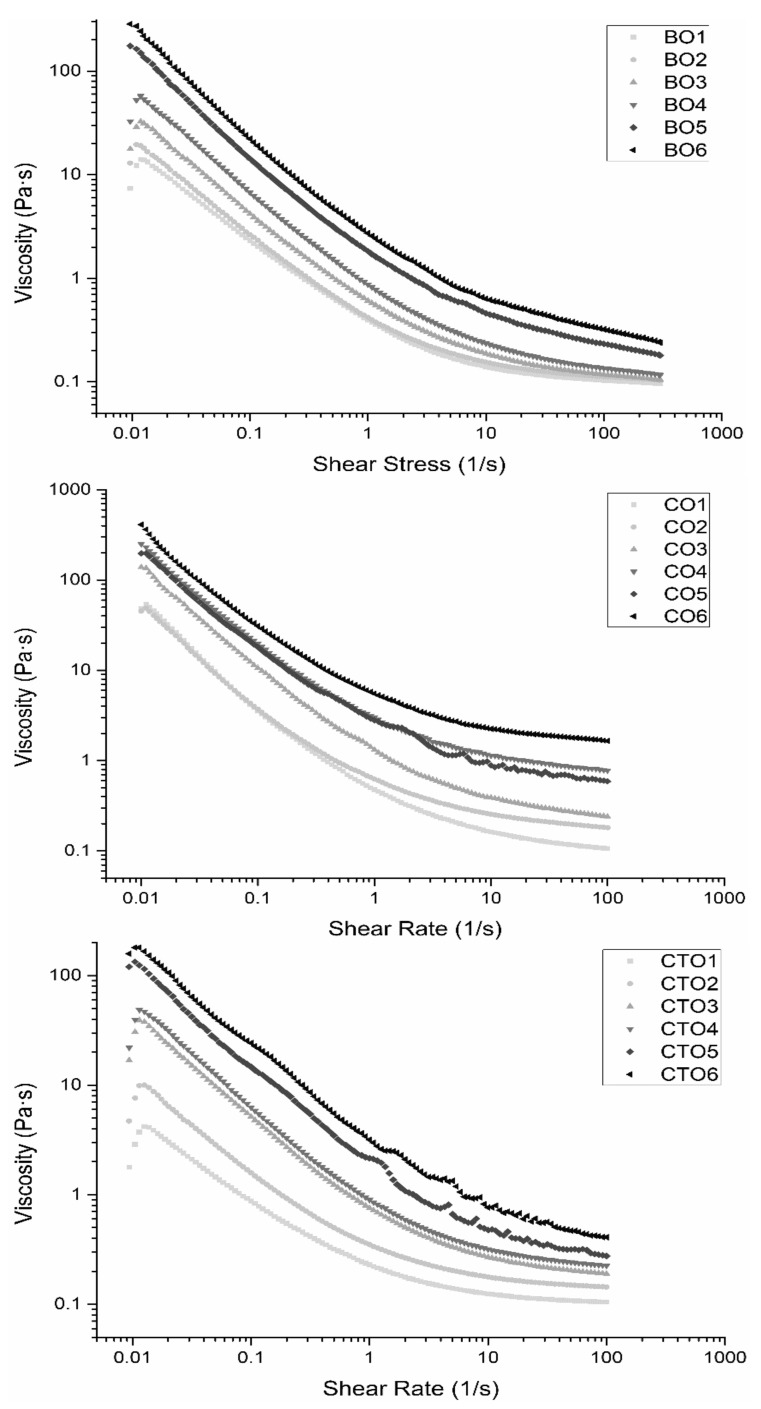
Flow curves for all organogel samples (up to 100 1/s).

Flow curves for all organogels’ samples can be seen in Figure 5. Results showed the behaviour of the samples without prior shearing at the transient state and at the steady state. It is possible to see that with an increase in gelator concentration there was an increase in shear stress throughout the shear rate interval (and, consequently, in viscosity) for all the different gelators tested. Such an increase can be related to the well-organized structure (that is stronger and more well organized the higher the concentration of wax). Static yield stress behaviour was also observed from stress vs. shear rate data of the 1st curve and measured as the initial stress required for the sample to flow (Table S1). These values also increased with wax concentration, not dependent on the gelator. Higher values could be related to more organized networks and/or stronger structures. This structure was broken with shear, reflecting a higher thixotropy. Table 1 shows the values of initial viscosity (low shear rate (0.05 s^−1^) and thixotropy. It is possible to see that the same trend as seen regarding initial viscosity and thixotropy is also found for yield stress, as samples reach higher yield stress values at higher concentrations.

Thixotropy, yield stress and initial viscosity were used as an indirect measure of strength and structure build-up of the organogel samples. For increasing gelator concentrations, stronger three-dimensional networks are formed by aggregation of the gelator crystals, and thus, stronger structures are expected, which is confirmed by the results seen in Table 1 [7,38]. Rheological results are also in agreement with textural measurements (Figure 4), where higher values for all mechanical properties were obtained for higher gelators’ concentration. For these tests, CO organogels displayed the highest values, thus being the one with the most well-defined structure and higher 3D network strength.

Organogels with higher concentrations of gelator, in part due to a stronger structure, exhibited higher standard deviations. Despite these high standard deviations, it was still possible to obtain the desired information on how the increase in concentration would affect the structure and, consequently, the thixotropy and initial viscosity. The same trend seen in the textural analyses was found in these thixotropy, viscosity and yield stress measurements, as candelilla produced the highest results (i.e., strongest gels) followed by beeswax and carnauba gels, indicating the same trend of higher HCC waxes producing organogels with stronger 3D structures. These trends were also seen in the microscopical and textural analysis of the organogels.

**Figure 6:**
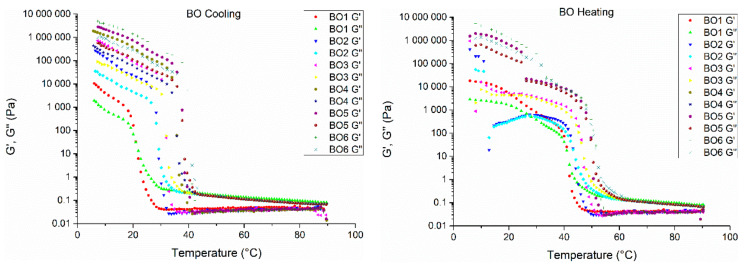

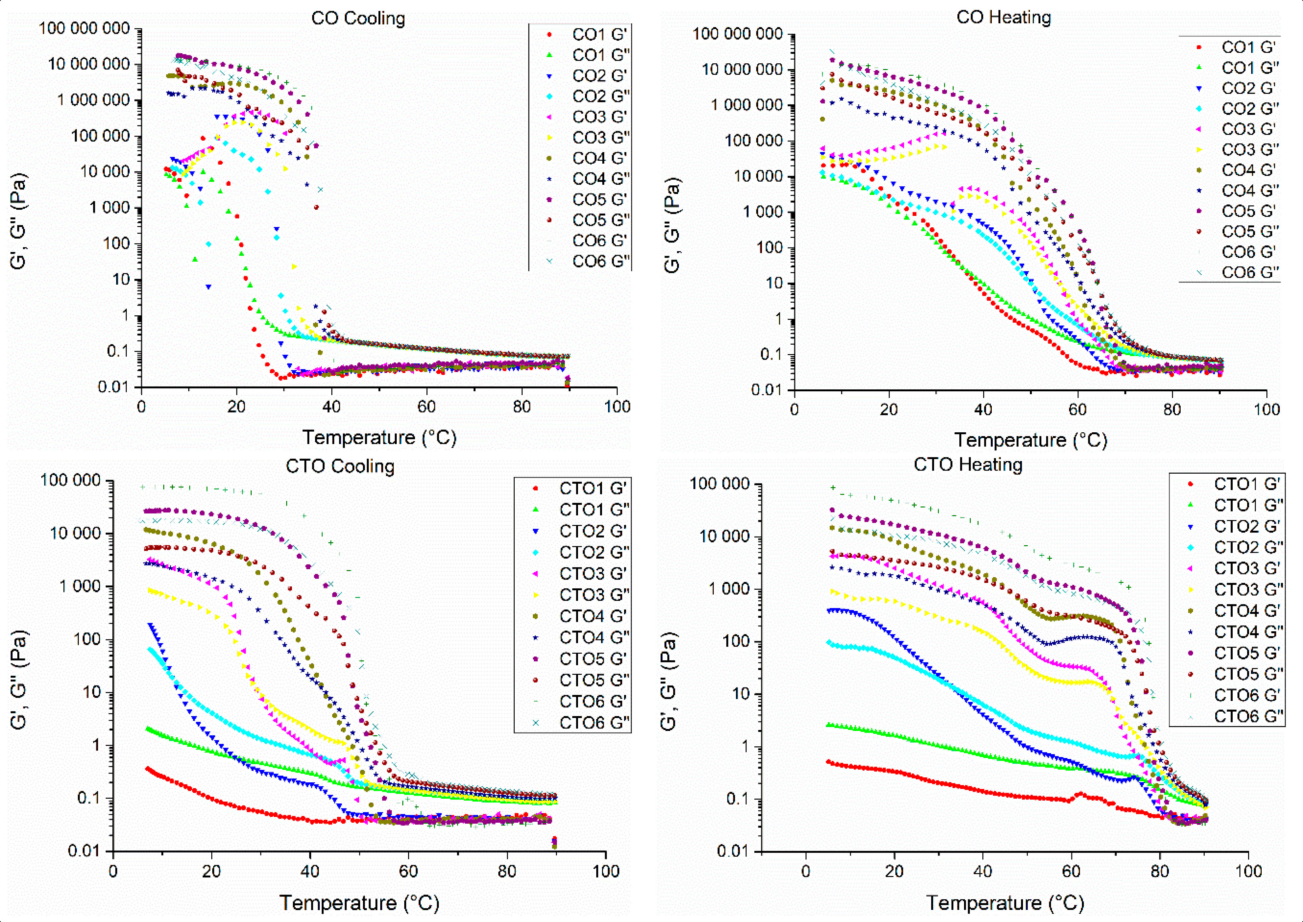
Temperature sweeps, heating and cooling stages are shown for all organogel samples. *G’*—Storage Modulus, *G’’*—Loss Modulus.

Non-isothermal rheology provided information on how gelator concentration affects the crystallization and melting processes of olive oil organogels. Samples were analysed between the regions of 90 and 5 °C, where it is possible to see at least two plateaus and at least one abrupt transition. Some samples displayed a second transition, of a lower slope, corresponding to a second phase transition of the organogels. The abrupt transition, seen in Figure 6, signals the rapid structural changes occurring during cooling or heating processes from liquid to solid organogel (or vice-versa) and represents the main crystallization or melting event, while a second transition of a lower slope (a second crystallization or melting event) signals the crystal network rearrangement to their final state, as seen previously in the DSC results (Table S2). Some samples showed more than two melting or crystallization processes but can easily be grouped within two major events. This is the case for some CTO organogels, where four melting events can be seen, two of them between 78.74–62.67 °C, and the other two between 44.30–20.78 °C. In these cases, only the major events were considered when comparing results between samples.

This multi-stage crystallization and melting process can be explained by the heterogeneous composition of the gelators, as well as the fact that these types of processes are time-dependent and happen stepwise (crystallization and melting, subsequently).

These results are also substantiated by the results seen in Table S2, where it is possible to see multiple melting and crystallization peaks. The multi-composition is related to the variation of the chemical composition between different waxes (mainly hydrocarbon, wax ester, fatty acid and fatty alcohol content), and as such, the gelation mechanism is a complicated dynamic that is reliant on a multi-composition containing multi-gelators.

Wax-based organogels’ melting and crystallization events are typically broader and occur shifted to lower temperature values than those of the corresponding neat waxes (please see Table S2). This is a result of the dilution of neat wax that occurs to form organogels at low gelator concentrations [29]. This dilution effect also results in having the predominant components present in the neat waxes govern the melting and crystallization behaviour of the organogels. For waxes with multiple components, this will lead to co-crystallization events [29].

Beeswax organogels (as seen in Table S2) have two main crystallization events, one more intense event around 40 °C, due to the wax esters, which are the main chemical component in beeswax (70% of its composition, mainly C16:0 for the fatty acid moieties, while the fatty alcohol moieties of the wax esters is constituted mostly by C16 to C32), and a second event of lower intensity around 25 °C, due to the HCC, the second major chemical component present in beeswax (around 15%, composed mainly by C27, C29 and C31). Candelilla wax has a more balanced composition between hydrocarbons (around 50%, mainly C31) and wax esters (around 40%, with fatty acids moieties composed mainly by C16, C18 and C22, and fatty alcohols moieties composed mainly of C18, C28 and C30) and displays one major crystallization event around 35 °C (probably due to its HCC), with another crystallization event at slightly higher temperatures, c.a. 40 °C, probably due to its wax ester content. Carnauba wax is mostly constituted by wax esters (around 60%, fatty acids moieties composed of C16 to C24, and the fatty alcohol moieties composed mostly of C18, C30 and C32) and free fatty alcohols (around 30%, mostly composed of C32), resulting in one major crystallization event around 55 °C due to the wax esters, and one smaller crystallization event around 45 °C due to the presence of free fatty alcohols. Crystallization events are typically influenced by the polarity of the groups present in the waxes’ chemical composition, while regarding the melting events, they are mainly influenced by the non-covalent interactions (e.g., such as London dispersion forces) that are formed during the molecular self-assembly of the organogels during gel formation [29,39].

From Figure 6 and from Table S2, it is possible to see that gelator concentration is the main factor responsible for the differences in the onset temperatures and state transition in the organogel samples for both the crystallization and melting stages events. By analysing these events, it is possible to see that temperatures increase with gelator concentration. Higher *G’* values are typically associated with stronger three-dimensional networks, which are formed by aggregation of the gelator crystals when the concentration of gelator is increased. A stronger three-dimensional network will need higher temperatures in order to be disentangled; therefore, higher melting points for organogels with higher concentrations of gelator are obtained [7,38].

It is also possible to compare the obtained data from rheological analyses with DSC measurements (Table S2). There are some differences between the two tables, which is to be expected given the different nature of the assessment. In DSC, thermal analyses data are obtained through measurements of energy changes at the molecular level, while in the non-isothermal oscillatory rheology, data are measured through changes in the bulk structure. In addition, in DSC analyses, organogel samples are already structured before analysis, while in the rheological analyses, samples undergo the crystallization process followed by the melting process during analysis. Results showed that organogels above their gelling point evidenced similar temperatures (except for the CO organogels), when compared to ones produced below the gelling point for each organogel (2, 1 and 3% (*w*/*w*), respectively, for BO, CO, and CTO). From Table S2, it is possible to see that melting temperature values obtained by DSC measurements are in the same range as those obtained from the rheological tests. As mentioned before, in some samples, melting and crystallization events occurred at different stages, which can be grouped into major events (typically of higher enthalpies and melting/crystallization temperatures).

These major events were used to compare the results obtained through DSC (peak melting and crystallization temperatures) and rheology techniques (sol–gel transition temperatures, determined through the derivation of the complex viscosity data).

A comparison between the obtained values for DSC and rheological analyses can be seen in Table S3. As shown by these results, the same behaviour can be seen in both measurements, and they typically occur within the same ranges. An increase in the values of assessed parameters is noticeable, for all organogels, as gelator concentration increases, showing the same behaviour in both measurements. Overall, for both heating and cooling stages, the evaluated rheological and thermal parameters are the highest for carnauba organogels, followed by candelilla and beeswax organogels, respectively, which is in accordance with suppliers’ information regarding melting temperature for the natural waxes.

Nevertheless, regarding *G’* and *G’’* values, candelilla organogels displayed the highest values, followed by beeswax and carnauba organogels, which can be related to lower mobility of gelator leading to a strengthened crystal network formed during the crystallization process. These results are in agreement with those seen from both the textural, flow rheology analysis and likely related to the HCC of the produced organogels, as candelilla organogels have the highest HCC, followed by beeswax and carnauba; the same trend is seen regarding both the *G’* and *G’’* values and the textural analysis results of the organogel samples.

## 3. Conclusions

Organogels showed changes in their properties when gelator concentration was increased, and when gelator type was changed. Oxidative stability was constant across all samples and well below both the values of both control samples and legal limits and, thus, is proper for use, even after a two-month storage period at industrial and/or commercial conditions. By comparing DSC and non-isothermal rheologic results, a clear tendency between melting and crystallization peaks and the increase in gelator concentration across the different types of gelators was shown.

Flow rheology assessed how the concentration of gelator and gelator type influenced the final characteristics of the organogel. Trends remained similar over different gelators, but overall, an increase in gelator concentration led to an increase in viscosity and in the force needed to disrupt the organogel structure. Information regarding the strength of the structure of the organogels was obtained through measurements of thixotropy and initial viscosity. It was clear that an increase in gelator concentration increased both the initial viscosity and thixotropy of the samples and, thus, created a stronger 3D network in the organogel, resulting in stronger structures. Beeswax organogels’ thixotropy increased from 770 to 44,491 Pa/s (1% to 6%), while initial viscosity increased from 26.75 to 1208 Pa·s (1% to 6%), while candelilla organogels increased from 840 to 34,104 Pa/s regarding thixotropy, and from 33.50 to 6785 Pa·s regarding initial viscosity, as concentration increased from 1 to 6%. Carnauba organogels followed the same tendency, increasing from 428 to 12,219 Pa/s and from 13 to 2504 Pa·s, regarding thixotropy and initial viscosity, respectively, from 1 to 6%. These data and conclusions are supported both by the microscopic analysis, where a more defined 3D crystal network is seen with the increase in gelator concentration, as well as by the textural results, which revealed a constant escalation in organogel firmness, spreadability and adhesiveness, as well as by the non-isothermal rheology results, with an increase in *G’* and *G’’* values for organogels of higher concentrations. It was also possible to observe that in these characterizations, candelilla organogels displayed superior strength values, followed by the beeswax and carnauba organogels. For example, regarding firmness, beeswax organogels displayed a maximum firmness of 5 N, while candelilla organogels displayed a maximum value of 17 N and carnauba organogels a maximum value of 4 N. Such results are indicative of a more organized 3D crystal network, possibly related to higher contents of hydrocarbons present in the natural composition. These changes in chemical composition (as well as the chain length of its components) of the natural waxes influenced the final characteristics of the developed organogels, as was shown mainly regarding the HCC of the natural waxes and how it correlated with the thermal, textural and rheological parameters analysed.

In all, these results show that by varying the gelator concentration and the gelator type, it is possible to obtain organogels with different characteristics and modulate said characteristics and produce organogels that are within oxidation legal limits, even when exposed to light and room temperature.

## 4. Materials and Methods

### 4.1. Materials

Gelator compounds (different waxes) were kindly donated by Ceras Marti (Barcelona, Spain), and their main properties are shown in Table 2. The extra virgin olive oil used was of commercial origin (Azeite Gallo, Lisbon, Portugal) and was purchased at a local supermarket.

### 4.2. Organogel Preparation

Beeswax, candelilla wax and carnauba wax were used as organogelators to produce, respectively, BO, CO and CTO organogels. Organogels were prepared by solubilizing the waxes (1 up to 6% (*w*/*w*)) in extra virgin olive oil at 90 °C for 30 min to ensure full solubilization. Then, the mixture was left to cool to room temperature (≈21 °C ± 2 °C), overnight, before sample use for characterization.

Samples were named according to waxes and concentration used (e.g., BO1 means 1% (*w*/*w*) beeswax), while pure extra virgin olive oil was used as control sample and identified as OOC.

Preliminary visual evaluation was used to identify the formation of organogels as self-supported structures. Tubes containing the produced samples were inverted, and the samples that did not flow under gravity were called organogels. The same evaluation was carried out after two months to confirm the structure’s stability.

### 4.3. Phase-Contrast Microscopy

Phase-contrast micrographs were obtained using an inverted microscope (Leica DMI 3000B), with phase-contrast illumination coupled with a high-sensitivity camera LEICA DFC450C (Leica Microsistemas Lda., Lisbon, Portugal). All images were acquired using the LAS 4.7 software (Leica Microsistemas Lda., Lisbon, Portugal). Organogel samples were prepared, and after settling at room temperature, they were placed directly in the support with a cover glass and then observed under the microscope.

### 4.4. Oxidative Stability

The oxidative stability (peroxide value—PV) was assessed spectrophotometrically according to the International Dairy Federation (IDF) standard method with some modifications [40,41,42]. The calibration curve for the IDF standard method was determined as suggested by Shantha and Decker (1994). The PV of the organogels was evaluated for 2 months of storage at room temperature and exposed to light to mimic real-life storage conditions. Pure extra virgin olive oil was stored in the same conditions and used as control.

Initially the oil was extracted from the organogels through the addition of 0.2 mL of organogel to 1.5 mL of isooctane/isopropanol (3:2, *v*/*v*) solution, in triplicate. These samples were then vortexed three times for 10 s each and left to stand for 30 min. After standing, 0.2 mL of the upper solvent layer was collected and mixed with 2.8 mL of chloroform/methanol solution (7:3, *v*/*v*) in a glass tube and vortexed again (4 s). Ammonium thiocyanate solution (15 µL) was added, and samples were vortexed for a further 4 s. After the addition of 15 µL Iron (II) solution, samples were once again vortexed for 4 s, and after 5 min incubation at room temperature in dim light conditions, the absorbance of the samples was measured at 500 nm using a JASCO V-650 UV/VIS Spectrophotometer (Jasco, Pfungstadt, Germany), in a glass cuvette, against a blank containing all the reagents except for the sample.

### 4.5. Mechanical Properties

All textural experiments were performed using a double axis texture analyser (Stable Microsystems, Surrey, UK) with a TTC Spreadability Rig (HDP/SR) probe consisting of a set of conical male (positive) and female (negative) acrylic 90° cones. All samples were placed into the sample holders and left to set at room temperature overnight. The positive cone was positioned 25 mm over the bottom of the lower cone and moved down 23 mm at 3 mm/s. Then, the probe returned to the initial position at 10 mm/s. Three samples from each formulation were evaluated at room temperature (23 °C). Firmness, adhesiveness and spreadability (maximum force (first maximum peak), negative work of shear (negative area) and positive work of shear (second positive area), respectively) were evaluated using the Texture Exponent v.6.1.1.0 software by Stable Microsystems (Surrey, UK). Each sample was assessed in triplicate. Pure extra virgin olive oil and commercial butter were used as control samples.

### 4.6. Thermal Analysis

Calorimetric studies were performed in a Perkin Elmer DSC 4000 differential scanning calorimeter (Perkin Elmer, Waltham, MA, USA). A DSC aluminium pan (B0143016) containing the organogel sample (ca. 5 mg) was placed in the DSC oven. An empty pan was used as reference. Samples were placed inside the DSC oven at room temperature, cooled to 5 °C (holding time of 1 min) and then analysed at 10 °C/min from 5 to 90 °C followed by a cooling curve to 5 °C at the same rate, under a nitrogen atmosphere. Melting and crystallization temperature peaks (Tm, Tc), their onset temperatures (Onset Tm, Onset Tc) and enthalpy changes (ΔHm, ΔHc) were calculated using Pyris software version 11.1 (Perkin Elmer, Waltham, MA, USA).

ΔG = ΔHc − TcΔS (1)

The entropy change (ΔS) can be calculated from Equation (1), assuming that at the crystallization temperature, the Gibb’s free energy tends to zero [27], where ΔG is the Gibb’s free energy change, and ΔS is the entropy change during crystallization, and ΔHc is the enthalpy change during crystallization, and Tc is the peak temperature during crystallization.

### 4.7. Rheological Analysis

Rheological measurements were carried out using a Discovery Hybrid Rheometer (DHR) (TA Instruments, NewCastle, DE, USA) and a stainless-steel plate geometry (40 mm diameter, 500 µm gap). Flow curves were obtained at 25 °C by an up (1st curve)-down (2nd curve)-up (3rd curve) step program with shear rate ranging from 0.01 to 300 s^−1^. The apparent viscosity at low shear rate (0.05 s^−1^—η0.05) was evaluated from unsteady-state (1st curve) to assess the less disturbed condition. Thixotropy was evaluated from hysteresis area between unsteady-state and steady-state flow curves (first and third, respectively). The area beneath the first and third curves was calculated using the equipment software (Trios v4.1.1.33073, New Castle, DE, USA), and qualitative thixotropy (Pa·s) was calculated subtracting the area under the first and third curves.

Non-isothermal oscillatory measurements were performed within the linear viscoelasticity region (0.1% strain, previously tested by strain sweep and not shown). The samples were transferred onto the rheometer plate, which was preheated at 90 °C. A cooling ramp was carried out between 90 and 5 °C at 10 °C min^−1^ with a fixed frequency of 1 Hz, followed by a heating ramp in the same conditions. Storage modulus G’ (elastic) and the loss modulus G’’ (viscous) were evaluated. Changes in the slope of complex viscosity (η*) as a function of temperature were evaluated from the derivatives of the data and used to better visualize and determine the sol–gel thermal transitions for both cooling and melting steps. Three replicates of each organogel sample were recorded for every rheological test.

### 4.8. Statistical Analyses

Statistical analyses were performed using analysis of variance, Tukey’s mean comparison test (*p* < 0.05) from results and conveyed as an average and standard deviation, using STATISTICA version 12.5 (StatSoft Inc., Tulsa, OK, USA, 2014).

## Figures and Tables

**Table 1:** Thixotropy and initial viscosity of all organogel samples. Different small letters indicate statistically significant differences between different concentrations within the same organogelator, while different capital letters indicate statistically significant differences between different organogels (*p* < 0.05).

Sample	Thixotropy (Pa/s)	Initial Viscosity (Pa·s)
BO1	769.7 ± 107.4 ^aA^	26.75 ± 1.81 ^aA^
BO2	1913 ± 161.9 ^aA^	195.2 ± 35.92 ^aA^
BO3	3290 ± 192.1 ^aA^	525.8 ± 93.70 ^abA^
BO4	7909 ± 2553 ^aA^	930.3 ± 233.1 ^bcA^
BO5	11,470 ± 1000 ^aA^	1154 ± 214.8 ^cA^
BO6	44,491 ± 8722 ^bA^	1208 ± 215.2 ^cA^
CO1	839.8 ± 58.40 ^aA^	33.60 ± 5.21 ^aA^
CO2	3963 ± 1417 ^abA^	268.9 ± 147.2 ^aA^
CO3	14,224 ± 1393 ^abB^	2703 ± 611.3 ^bB^
CO4	16,516 ± 5553 ^abA^	4722 ± 857.4 ^cB^
CO5	28,613 ± 23,177 ^abA^	5945 ± 610.0 ^cdB^
CO6	34,104 ± 121.3 ^bA^	6785 ± 403.0 ^dB^
CTO1	427.5 ± 11.80 ^aB^	12.78 ± 1.51 ^aB^
CTO2	1264 ± 36.16 ^aB^	132.2 ± 9.40 ^abA^
CTO3	2324 ± 20.00 ^aA^	345.1 ± 51.57 ^abA^
CTO4	3444 ± 299.2 ^aB^	1095 ± 4.26 ^bA^
CTO5	6238 ± 2625 ^aA^	2510 ± 459.4 ^cA^
CTO6	12,219 ± 5390 ^bB^	2504 ± 542.8 ^cC^

**Table 2:** Gelator properties.

Natural Waxes	Melting Point (°C)	Acidity Value (mg KOH/g)	Saponification Value (mg KOH/g)	Ester Content (mg KOH/g)
Beeswax	61–65	14–24	82–104	70–80
Candelilla Wax	69–73	12–22	43–63	31–43
Carnauba Wax	80–86	02–07	78–95	71–88

## Data Availability

Data is contained within the article or supplementary material.

## Supplementary Materials

The following are available online at https://www.mdpi.com/2310-2861/7/1/12/s1, Table S1: Yield stress of all organogel samples; Table S2: Thermal properties of organogels (temperature of melting—*T_m_*; enthalpy of melting—Δ*H_m_*; temperature of crystallization—*T_c_*; enthalpy of crystallization—Δ*H_c_*; entropy of crystallization—Δ*S_c_*; sol–gel temperature transition during the melting—T*_g,melting_*; sol–gel temperature transition during the cooling—T*_g,cooling_*); Table S3: Comparison of melting and crystallization temperatures obtained in DSC and rheological analysis.
