# Supplementary material for: Modulation and Characterization of Wax-Based Olive Oil Organogels in View of Their Application in the Food Industry
Source: Gels. 2021 Jan 28;7(1):12. doi: 10.3390/gels7010012 (PMC7931099; doi:10.3390/gels7010012)
Supplement: Supplementary file 1 [file gels-07-00012-s001.pdf]

*Supplementary Materials*

# Modulation and Characterization of Wax-Based Olive Oil Organogels in View of Their Application in the Food Industry

**Pedro M. Silva [1,2], Artur J. Martins [2], Luiz H. Fasolin [3] and António A. Vicente [1,*]**

[1] Centre of Biological Engineering, University of Minho, Campus de Gualtar, 4710-057 Braga, Portugal; pedro.silva@ceb.uminho.pt
[2] Food Processing and Nutrition Group, International Iberian Nanotechnology Laboratory, Av. Mestre José Veiga s/n, 4715-330 Braga, Portugal; artur.martins@inl.int
[3] Department of Food Engineering, School of Food Engineering, University of Campinas—UNICAMP, 13083-862 Campinas, SP, Brazil; lfasolin@unicamp.br
* Correspondence: avicente@deb.uminho.pt

**Table S1.** Static yield stress of all organogel samples. Different small letters indicate statistically significant differences between different concentrations within the same organogelator, while different capital letters indicate statistically significant differences between different organogels ($p<0.05$).

| Sample | Yield Stress (Pa) |
|---|---|
| BO1 | $5.1 \pm 1.92^{aA}$ |
| BO2 | $28.58 \pm 0.43^{aA}$ |
| BO3 | $91.3 \pm 31.91^{aA}$ |
| BO4 | $221.09 \pm 72.97^{abA}$ |
| BO5 | $545.59 \pm 280.23^{bcA}$ |
| BO6 | $803.56 \pm 166.05^{cA}$ |
| CO1 | $3.77 \pm 0.7^{aA}$ |
| CO2 | $35.25 \pm 21.51^{aA}$ |
| CO3 | $418.65 \pm 102.2^{abB}$ |
| CO4 | $832.09 \pm 513.32^{abA}$ |
| CO5 | $1395.57 \pm 369.35^{abB}$ |
| CO6 | $1824.94 \pm 1081.63^{bA}$ |
| CTO1 | $0.07 \pm 0.03^{aB}$ |
| CTO2 | $9.95 \pm 0.69^{abA}$ |
| CTO3 | $30.28 \pm 8.16^{abA}$ |
| CTO4 | $94.84 \pm 7.33^{abA}$ |
| CTO5 | $218.73 \pm 73.88^{abA}$ |
| CTO6 | $409.33 \pm 353.72^{bA}$ |

**Table S2** Thermal properties of organogels (temperature of melting—$T_m$; enthalpy of melting—$\Delta H_m$; temperature of crystallization—$T_c$; enthalpy of crystallization—$\Delta H_c$; entropy of crystallization—$\Delta S_c$; sol–gel temperature transition during the melting—$T_{g,melting}$; sol–gel temperature transition during the cooling—$T_{g,cooling}$)

| Sample | Onset $T_{m1}$ (°C) | $T_{m1}$ (°C) | Endset $T_{m1}$ (°C) | $\Delta H_{m1}$ (J/g) | Onset $T_{c1}$ (°C) | $T_{c1}$ (°C) | Endset $T_{c1}$ (°C) | $\Delta H_{c1}$ (J/g) | $\Delta S_{c1}$ (J/g) | Onset $T_{m2}$ (°C) | $T_{m2}$ (°C) | Endset $T_{m2}$ (°C) | $\Delta H_{m2}$ (J/g) | Onset $T_{c2}$ (°C) | $T_{c2}$ (°C) | Endset $T_{c2}$ (°C) |
|---|---|---|---|---|---|---|---|---|---|---|---|---|---|---|---|---|
| BO1 | 21.47 | 25.64 | 25.64 | 0.04 | 30.57 | 28.03 | 23.80 | -0.49 | -0.02 | 32.93 | 42.64 | 49.64 | 0.65 | N.D. | N.D. | N.D. |
| BO2 | 22.73 | 25.07 | 25.07 | 0.02 | 36.68 | 32.59 | 30.97 | -1.21 | -0.04 | 34.65 | 46.29 | 53.27 | 1.80 | 23.47 | 18.49 | 9.75 |
| BO3 | 15.84 | 19.73 | 24.01 | 0.05 | 39.86 | 36.77 | 31.69 | -1.86 | -0.05 | 28.45 | 27.01 | 28.29 | 0.24 | 26.70 | 22.39 | 14.16 |
| BO4 | 13.84 | 18.12 | 23.56 | 0.07 | 41.85 | 39.05 | 34.17 | -2.30 | -0.06 | 24.28 | 27.84 | 31.13 | 0.20 | 28.48 | 24.38 | 15.58 |
| BO5 | 15.05 | 20.22 | 22.22 | 0.14 | 42.84 | 40.62 | 36.75 | -3.06 | -0.08 | 27.59 | 30.11 | 31.64 | 0.33 | 29.79 | 26.11 | 18.26 |
| BO6 | 16.72 | 22.94 | 26.36 | 0.13 | 44.37 | 41.95 | 38.15 | -3.67 | -0.09 | 29.01 | 30.28 | 31.96 | 0.25 | 30.87 | 26.94 | 19.36 |
| CO1 | 21.93 | 25.14 | 27.40 | 0.04 | 28.49 | 26.68 | 24.57 | -0.10 | 0.00 | 33.98 | 39.98 | 43.91 | 0.22 | 23.50 | 19.96 | 12.90 |
| CO2 | 21.11 | 25.77 | 28.00 | 0.09 | 34.08 | 32.09 | 30.09 | -0.26 | -0.01 | 30.50 | 32.17 | 34.01 | 0.13 | 29.39 | 26.01 | 19.15 |
| CO3 | 19.99 | 26.18 | 28.97 | 0.26 | 36.73 | 35.88 | 34.09 | -0.59 | -0.02 | 31.02 | 34.26 | 36.06 | 0.33 | 33.41 | 30.66 | 24.31 |
| CO4 | 19.93 | 26.42 | 29.54 | 0.29 | 39.16 | 37.93 | 36.49 | -0.71 | -0.02 | 32.80 | 43.42 | 50.32 | 3.93 | 35.84 | 32.88 | 29.67 |
| CO5 | 21.65 | 25.26 | 27.54 | 0.07 | 40.64 | 39.35 | 37.95 | -0.76 | -0.02 | 36.66 | 44.14 | 51.74 | 4.82 | 37.41 | 34.63 | 29.57 |
| CO6 | 22.18 | 25.40 | 28.45 | 0.13 | 41.26 | 40.15 | 38.77 | -1.21 | -0.03 | 33.33 | 44.34 | 50.20 | 6.02 | 38.20 | 35.49 | 30.39 |
| CTO1 | 37.18 | 43.33 | 48.08 | 0.07 | 44.63 | 42.06 | 38.74 | -1.07 | -0.03 | 65.10 | 70.82 | 76.91 | 0.95 | 23.22 | 19.23 | 12.44 |
| CTO2 | 39.68 | 46.31 | 50.06 | 0.09 | 45.93 | 42.52 | 36.78 | -1.01 | -0.02 | 66.10 | 72.09 | 78.23 | 1.01 | 24.65 | 20.02 | 12.76 |
| CTO3 | 41.40 | 49.81 | 53.89 | 0.20 | 50.13 | 47.58 | 42.92 | -2.60 | -0.05 | 67.84 | 73.64 | 80.22 | 2.41 | 30.24 | 26.08 | 19.16 |
| CTO4 | 42.56 | 50.79 | 55.14 | 0.49 | 53.90 | 50.32 | 43.80 | -4.78 | -0.10 | 68.98 | 74.73 | 80.89 | 4.71 | 34.94 | 30.81 | 22.32 |
| CTO5 | 44.08 | 52.24 | 56.06 | 0.64 | 58.37 | 54.81 | 50.36 | -6.15 | -0.11 | 69.17 | 75.28 | 81.23 | 8.26 | 49.18 | 47.29 | 45.35 |
| CTO6 | 45.26 | 52.62 | 57.31 | 0.47 | 58.24 | 54.49 | 48.75 | -5.94 | -0.11 | 70.13 | 76.72 | 81.35 | 8.96 | 48.27 | 45.99 | 43.08 |
| Control BW | 63.22 | 67.80 | 69.84 | 140.83 | 60.60 | 58.51 | 50.80 | -85.52 | -1.46 | N.D. | N.D. | N.D. | N.D. | 50.22 | 48.36 | 45.00 |
| Control CW | 68.07 | 73.67 | 76.55 | 116.52 | 68.93 | 66.59 | 63.84 | -7.02 | -0.11 | N.D. | N.D. | N.D. | N.D. | 62.82 | 60.03 | 56.33 |
| Control CT1W | 52.58 | 59.37 | 63.37 | N.D. | 78.87 | 75.96 | 71.31 | -84.84 | -1.12 | 82.30 | 85.39 | 88.03 | 125.44 | 70.69 | 68.65 | 64.28 |

**Table S2 (continuation)** Thermal properties of organogels (temperature of melting—$T_m$; enthalpy of melting—$\Delta H_m$; temperature of crystallization—$T_c$; enthalpy of crystallization—$\Delta H_c$; entropy of crystallization—$\Delta S_c$; sol–gel temperature transition during the melting—$T_{g,melting}$; sol–gel temperature transition during the cooling—$T_{g,cooling}$)

| Sample | $\Delta H_{c2}$ (J/g) | $\Delta S_{c2}$ (J/g) | Onset $T_{m3}$ (°C) | $T_{m3}$ (°C) | Endset $T_{m3}$ (°C) | $\Delta H_{m3}$ (J/g) | Onset $T_{c3}$ (°C) | $T_{c3}$ (°C) | Endset $T_{c3}$ (°C) | $\Delta H_{c3}$ (J/g) | $\Delta S_{c3}$ (J/g) | $T_{g1}$ Melting | $T_{g2}$ Melting | $T_{g3}$ Melting | $T_{g4}$ Melting | $T_{g1}$ Cooling | $T_{g2}$ Cooling |
|---|---|---|---|---|---|---|---|---|---|---|---|---|---|---|---|---|---|
| BO1 | N.D. | N.D. | N.D. | N.D. | N.D. | N.D. | N.D. | N.D. | N.D. | N.D. | N.D. | 41.21 | 23.28 | N.D. | N.D. | 21.01 | N.D. |
| BO2 | -0.51 | -0.03 | N.D. | N.D. | N.D. | N.D. | N.D. | N.D. | N.D. | N.D. | N.D. | 43.88 | 16.74 | N.D. | N.D. | 29.56 | 14.43 |
| BO3 | -1.13 | -0.05 | 39.33 | 48.61 | 54.80 | 4.32 | N.D. | N.D. | N.D. | N.D. | N.D. | 41.21 | 22.68 | N.D. | N.D. | 21.01 | N.D. |
| BO4 | -1.44 | -0.06 | 36.50 | 49.67 | 55.76 | 4.46 | N.D. | N.D. | N.D. | N.D. | N.D. | 50.27 | 23.29 | N.D. | N.D. | 36.62 | 21.76 |
| BO5 | -2.16 | -0.08 | 38.56 | 51.05 | 49.88 | 6.72 | N.D. | N.D. | N.D. | N.D. | N.D. | 50.33 | 32.31 | N.D. | N.D. | 39.17 | 24.87 |
| BO6 | -2.71 | -0.10 | 39.96 | 51.88 | 56.94 | 7.04 | N.D. | N.D. | N.D. | N.D. | N.D. | 50.85 | 30.49 | N.D. | N.D. | 40.63 | 26.02 |
| CO1 | -0.29 | -0.01 | 51.00 | 57.86 | 61.76 | 0.12 | N.D. | N.D. | N.D. | N.D. | N.D. | 33.35 | 18.80 | N.D. | N.D. | 20.73 | 9.91 |
| CO2 | -0.93 | -0.04 | 35.23 | 41.83 | 50.26 | 1.62 | N.D. | N.D. | N.D. | N.D. | N.D. | 49.95 | 19.67 | N.D. | N.D. | 28.41 | 10.28 |
| CO3 | -2.21 | -0.07 | 36.71 | 42.34 | 50.29 | 4.01 | N.D. | N.D. | N.D. | N.D. | N.D. | 55.58 | 32.31 | N.D. | N.D. | 32.06 | 11.02 |
| CO4 | -2.94 | -0.09 | 58.77 | 62.26 | 67.07 | 1.57 | N.D. | N.D. | N.D. | N.D. | N.D. | 59.94 | 46.66 | N.D. | N.D. | 35.93 | 10.46 |
| CO5 | -3.16 | -0.09 | 58.78 | 63.64 | 68.22 | 2.23 | N.D. | N.D. | N.D. | N.D. | N.D. | 64.08 | 47.86 | N.D. | N.D. | 38.16 | 11.69 |
| CO6 | -5.15 | -0.15 | 56.27 | 62.12 | 69.13 | 3.69 | N.D. | N.D. | N.D. | N.D. | N.D. | 64.47 | 48.87 | N.D. | N.D. | 39.40 | 12.93 |
| CTO1 | -0.09 | 0.00 | N.D. | N.D. | N.D. | N.D. | N.D. | N.D. | N.D. | N.D. | N.D. | 78.37 | 62.67 | 38.95 | 23.25 | 43.44 | N.D. |
| CTO2 | -0.12 | -0.01 | N.D. | N.D. | N.D. | N.D. | N.D. | N.D. | N.D. | N.D. | N.D. | 77.71 | 63.88 | 46.07 | 20.78 | 46.79 | N.D. |
| CTO3 | -0.30 | -0.01 | N.D. | N.D. | N.D. | N.D. | N.D. | N.D. | N.D. | N.D. | N.D. | 78.74 | 66.39 | 44.30 | 23.58 | 50.47 | 39.98 |
| CTO4 | -0.44 | -0.01 | N.D. | N.D. | N.D. | N.D. | N.D. | N.D. | N.D. | N.D. | N.D. | 73.68 | 48.16 | N.D. | N.D. | 52.40 | 34.50 |
| CTO5 | -2.38 | -0.05 | N.D. | N.D. | N.D. | N.D. | 38.97 | 36.12 | 29.64 | -0.72 | -0.02 | 77.43 | 48.43 | N.D. | N.D. | 48.31 | 39.25 |
| CTO6 | -2.47 | -0.05 | N.D. | N.D. | N.D. | N.D. | 37.82 | 34.43 | 28.68 | -0.64 | -0.02 | 78.40 | 48.32 | N.D. | N.D. | 62.76 | 50.47 |
| Control BW | -56.45 | -1.17 | N.D. | N.D. | N.D. | N.D. | N.D. | N.D. | N.D. | N.D. | N.D. | | | | | | |
| Control CW | -68.60 | -1.14 | N.D. | N.D. | N.D. | N.D. | 55.01 | 53.44 | 51.14 | -37.80 | -0.71 | | | | | | |
| Control CT1W | -52.67 | -0.77 | N.D. | N.D. | N.D. | N.D. | N.D. | N.D. | N.D. | N.D. | N.D. | | | | | | |

**Table S3** Comparison of melting and crystallization temperatures obtained in DSC and rheological analysis

| Sample | Tm1 (°C) | Tg1 Melting (°C) | Tm2 (°C) | Tg2 Melting (°C) | Tc1 (°C) | Tg1 Cooling (°C) | Tc2 (°C) | Tg2 Cooling (°C) |
|---|---|---|---|---|---|---|---|---|
| BO1 to BO6 | 18.12 to 25.64 | 16.74 to 32.31 | 42.64 to 51.88 | 41.21 to 50.85 | 23.47 to 30.87 | 14.43 to 26.02 | 28.03 to 41.85 | 21.01 to 40.63 |
| CO1 to CO6 | 39.98 to 44.34 | 18.80 to 48.87 | 57.86 to 62.12 | 33.35 to 64.47 | 19.96 to 35.49 | 9.91 to 12.93 | 26.68 to 40.15 | 20.73 to 39.40 |
| CTO1 to CTO6 | 43.33 to 52.72 | 38.95 to 48.43 | 70.82 to 76.72 | 73.68 to 78.74 | 19.23 to 47.29 | 39.98 to 50.47 | 42.06 to 54.49 | 43.44 to 62.76 |
